# An iris puncture technique for restoration of the anterior chamber in vitrectomy for pediatric vitreoretinopathies

**DOI:** 10.3389/fped.2022.961379

**Published:** 2022-09-02

**Authors:** Chunli Chen, Feng Hu, Tian Tian, Yizhe Cheng, Ping Fei, Peiquan Zhao

**Affiliations:** ^1^Department of Ophthalmology, Xinhua Hospital Affiliated to Shanghai Jiaotong University, Shanghai, China; ^2^Department of Ophthalmology, Beijing Tongren Hospital, Capital Medical University, Beijing, China; ^3^Beijing Ophthalmology and Visual Science Key Laboratory, Beijing, China

**Keywords:** iris puncture, anterior chamber restoration, vitrectomy, pediatric vitreoretinopathies, surgical methods

## Abstract

**Purpose:**

To present a novel usage of iris puncture-assisted lensectomy with anterior vitrectomy or vitrectomy in pediatric patients with the absence of anterior chamber caused by various advanced vitreoretinopathies complicated with capsule-endothelial, iris-endothelial adhesion, and secondary glaucoma.

**Design:**

Prospective study.

**Materials and methods:**

Forty-one patients were enrolled in this consecutive, prospective study. The iris puncture was performed in all patients using a 20G Vitrectomy Microsurgical Knife, followed by the lensectomy with anterior vitrectomy or vitrectomy. Demographic information, the number of iris puncture times, surgical procedure, intraoperative and postoperative complications, therapy, and prognosis were collected. Patients were followed up for at least 6 months.

**Results:**

A total of 18 female patients and 23 male patients were included, with a mean age of 9.5 ± 7.5 months. The formation of anterior chamber formation was achieved in 28 (68.3%) eyes, with only 1 initial episode of iris puncture, 11 (26.8%) patients required 2 episodes, and 3 episodes of iris puncture, with additional external drainage of subretinal fluid, were needed in the remaining 2 (4.9%) patients. Except for iris incarceration, which occurred in 7 (17%) eyes during operation, there was no iridodialysis or subretinal fluid overflow during operation. At the last visit (mean: 12.16 ± 5.38 months of follow-up), all eyes had a reconstructed anterior chamber with normal depth. No synechiae between the iris and the cornea occurred after surgery. The mean postoperative intraocular pressure was 6.23 ± 1.64 mmHg. A hazy cornea vanished in 31 out of 41 (75.6%) eyes, relieved in 8 out of 41 eyes (19.5%), and 2 out of 41 eyes (4.88%) did not change. In the 25 eyes accepting vitrectomy and lensectomy, 20 out of 25 (80%) achieved different degrees of reattachment.

**Conclusion:**

The innovative iris puncture technique is effective, simple, and safe management for the anterior chamber disappearance caused by various advanced pediatric vitreoretinopathies, which helped to lower the intraocular pressure and offers a chance for lensectomy with anterior vitrectomy or vitrectomy.

## Introduction

The pediatric retinal detachment (RD) induced by advanced vitreoretinopathies is challenging for surgeons in planning surgical management. Compared with adults, pediatric RD is more often presented as macular involved detachment, proliferative vitreoretinopathy (PVR), chronic course, and worse visual acuity (1, 2). Besides, the etiology of pediatric RD is complex and heterogeneous. The most common vitreoretinopathies that can cause RD include familial exudative vitreoretinopathy (FEVR), Norrie disease, Coats disease, persistent hyperplastic primary vitreous (PHPV) syndrome, and retinopathy of prematurity (ROP).

With disease progression, advanced vitreoretinopathies may cause the absence of the anterior chamber (AC). If untreated, many complications might be brought about, such as secondary glaucoma, corneal degeneration, or even phthisis bulbi. To avoid these complications, lensectomy with anterior vitrectomy or vitrectomy is recommended. However, during the operation, the handling of anterior chamber formation can be really difficult. Traditionally, external drainage of subretinal fluid (SRF) was recommended and applied to form the anterior chamber in cases with a flat anterior chamber secondary to advanced pediatric vitreoretinopathies (3–5). However, external drainage may generate many serious complications, such as retinal incarceration, subretinal hemorrhage, iatrogenic retinal holes, and loss of vitreous, which are highly sight-threatening (6–9). The author’s previous experience has confirmed that a sudden and rapid outflow of SRF may also lead to iridodialysis. Besides, the constant flow of subretinal fluid could also reduce the definition of the surgical field. These disadvantages of external drainage of SRF cried out for a more effective and safer technique in the management of the disappeared AC in the pediatric population with advanced vitreoretinopathies. To solve these problems, we performed iris puncture instead of external drainage of SRF in pediatric patients with the absence of AC.

Over the past years, many pediatric patients with absent AC caused by various advanced vitreoretinopathies have been referred to the authors’ clinical center. We herein described innovative iris puncture techniques in these pediatric patients.

## Materials and methods

This study adhered to the tenets of the Declaration of Helsinki and was approved by the regional ethics committee. As the participants were all underage children, informed written consent was obtained from their parents or guardians. This study is a consecutive, prospective, and interventional case series.

### Patients

Forty-one patients (41 eyes) with absent anterior chamber induced by various advanced vitreoretinopathies (including 30 eyes with FEVR, 6 eyes with PHPV, and 5 eyes with ROP) were collected in the present study between January 2016 and October 2017. The surgical indications were the absent anterior chamber caused by various vitreoretinopathies. Patients were excluded if obvious iris neovascularization was observed. All 41 patients had complete ophthalmologic examinations including anterior and posterior examination by biomicroscope, B-scan (Digital B 2000 and Ultrascan Imaging System; Alcon), axial length by A-scan (Digital B 2000 and Ultrascan Imaging System; Alcon), intraocular pressure, and fundus photography by Retcam (Clarity Medical Systems, Pleasanton, CA). The follow-up period is at least 1 month.

### Surgical technique

**FIGURE 1:**
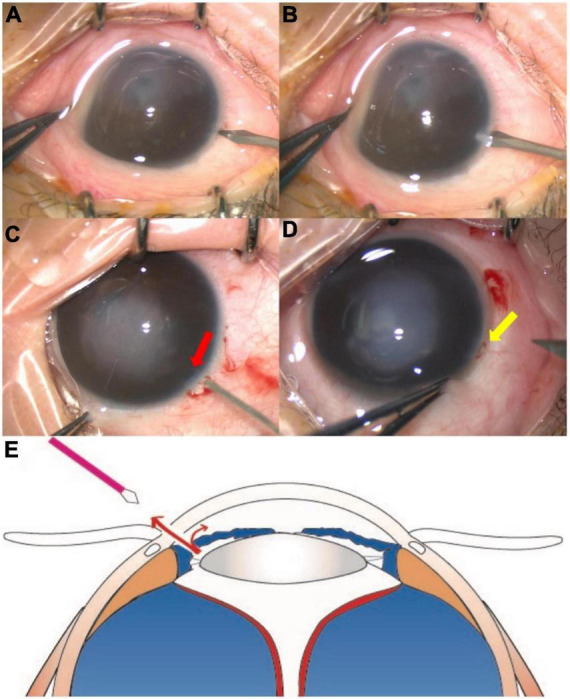
The major steps of the iris incision. The iris incision was performed using the 20G vitrectomy microsurgical knife through a corneal incision **(A,B)**. After the puncture, the iris incision (red arrow) was observed **(C)**. The posterior aqueous humor (yellow arrow) flowed out from the incision **(D)**. The diagram shows that the posterior chamber fluid flows into the anterior chamber through the 27G iris incision, and fluid in the anterior chamber also outflows through the incision on the corneal limbus **(E)**.

After the administration of general anesthesia, the procedure was performed under a surgical microscope. The main steps are shown in Figure 1 and Supplementary Material of the video. Iris puncture was performed using a 20G vitrectomy microsurgical knife through the corneal incision near limbus approaches. The punctuation site of the iris was vertically through the limbus, which is in the vicinity of the ciliary suspensory ligament. The outflow of posterior aqueous humor was observed after the first iris puncture of the corneal incision (Figure 1D). Generally, after the first iris puncture through corneal incision, the pressure of the posterior chamber could be partially released, and the anterior chamber could then be formed after the application of viscoelastic. However, in a few cases, if the anterior chamber formation could not be achieved with the assistance of a Healon agent after one episode of iris puncture, a second or third iris puncture would be performed through different corneal incision sites. The puncture incision(s) could still be used in further maneuvers; for example, we could insert the perfusion cannula from this incision with no fear of the occurrence of subretinal or suprachoroidal perfusion. The combination of external drainage of subretinal SRF was performed if the anterior chamber formation failed after three episodes of iris puncture. In the current study, the combination of SRF drainage was performed in two (4.8%) cases, in which SRF was drained out transclerally through a 27-gauge incision (generated by a 27-gauge penetrating needle) near the equator corresponding to the greatest retinal detachment height. After the formation of the anterior chamber with the assistance of a Healon agent, an infusion cannula connected to a balanced salt plus solution (Alcon, Laboratories, Inc.) was inserted into the anterior chamber through the corneal incision followed by the lensectomy with anterior vitrectomy or vitrectomy.

## Results

**FIGURE 2:**
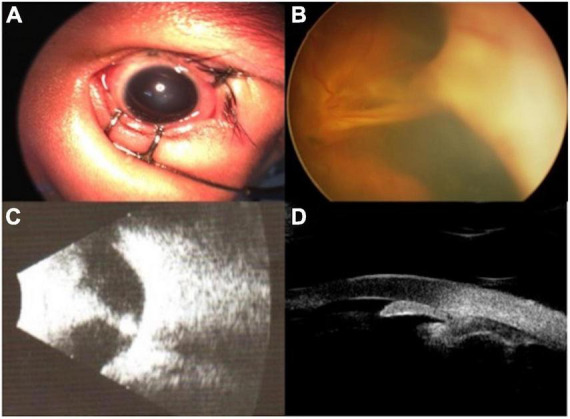
The preoperative external and fundus photograph, ultrasound, and UBM of typical patients. An external photograph showed corneal edema **(A)**. Fundus image showed extensive funnel-like retinal detachment **(B)**. Ocular ultrasound showed retinal detachment **(C)**. UBM showed the disappearance of the anterior chamber **(D)**.

The demographic and clinical characteristics are shown in Table 1. There were 18 females and 23 males, with a mean age of 9.5 ± 7.5 months. The novel technique of iris puncture was successfully performed in 41 eyes (41 patients) with an absent anterior chamber resulting from advanced vitreoretinopathies (30 FEVR, 6 PHPV, and 5 ROP). Typical patients are shown in Figure 2. The anterior chamber formation was achieved in 28 (68.3%) eyes with just 1 initial episode of iris puncture *via* corneal incision, 11 (26.8%) patients required 2 episodes, and 3 episodes of iris puncture combined with external SRF drainage were needed in the remaining 2 (4.9%). There was no iridodialysis or subretinal fluid overflow during the operation. The mean preoperative intraocular pressure (IOP) was 10.25 ± 4.21 mmHg. The mean postoperative IOP was 6.23 ± 1.64 mmHg, which is lower than preoperative ones with statistical significance (*P* = 0.02). At the last visit (mean:12.16 ± 5.38 months of follow-up), all eyes had a reconstructed AC. No synechiae between the iris and the cornea occurred after surgery. For patients over 3 years old, the AC depth was observed *via* a slip lamp. For patients under 3 years old, the AC depth was observed *via* RetCam examination and indirect ophthalmoscope. Additionally, for patients who need second intraocular lens implantation, the AC depth was observed *via* ultrasound biomicroscopy. No other related complications were noted at the last follow-up.

**TABLE 1:** Demographic information of patients.

Variable	No. Patients (%)
Gender (*N* = 41)	
Male	23 (56%)
Female	18 (44%)
Age (Mean ± SD)	9.5 ± 7.5 months
Etiology	
FEVR	30 (73%)
PHPV	6 (15%)
ROP	5 (12%)

FEVR, familial exudative vitreoretinopathy; ROP, retinopathy of prematurity;PHPV, persistent hyperplastic primary vitreous; SD, standard deviation.

**FIGURE 3:**
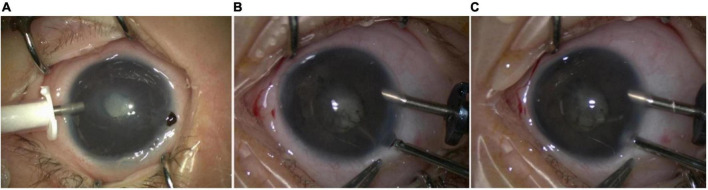
The intraoperative complication of iris incision. The major intraoperative complication of iris incision was iris incarceration **(A)**. The incarcerated iris can be easily returned to the anterior chamber with the assistance of surgical instruments or a viscoelastic agent **(B,C)**.

**FIGURE 4:**
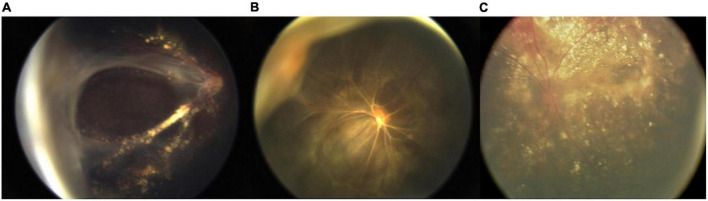
Different degrees of retina reattachment. Partial reattachment with posterior retina detached **(A)**. Major reattachment with peripheral retinal detached **(B)**. Total reattachment **(C)**.

The clinical characteristics and surgical methods applied in these procedures are shown in Table 2. The major intraoperative complication of iris puncture was iris incarceration, which occurred in 7 (17%) of the 41 eyes (Figure 3). Incarcerated iris was successfully returned in all 7 cases. Postoperatively, the anterior chamber remained deep and stable in all cases. No iris incarceration and other related complications were observed at the last follow-up. Hazy cornea vanished in 31 out of 41 (75.6%) eyes, relieved in 8 out of 41 eyes (19.5%), and 2 out of 41 eyes (4.88%) not changed. In the 25 eyes accepting vitrectomy and lensectomy, 20 out of 25 (80%) achieved different degrees of re-attachment (Figure 4).

**TABLE 2:** Demographic information and surgical procedure of study subjects.

No.	Sex	Age	Eye	Diagnosis	Puncture sites	Combine SRF drainage	Surgical procedure	Intraoperative complications	Postoperative complications
1	M	2 months	OD	FEVR	1	N	Lensectomy	None	None
2	F	1 year	OS	FEVR	2	N	Lensectomy	None	None
3	M	4 months	OS	FEVR	1	N	Lensectomy	Iris incarceration	None
4	F	4 months	OS	PHPV	2	N	Lensectomy	None	None
5	M	4 months	OD	ROP	1	N	Lensectomy	None	None
6	M	10 months	OD	FEVR	1	N	Lensectomy	None	None
7	F	8 months	OS	FEVR	2	N	Lensectomy	None	None
8	M	2 years	OS	FEVR	1	N	Lensectomy +Vtx	None	None
9	M	2 years	OD	FEVR	1	N	Lensectomy +Vtx	None	None
10	F	2 months	OS	PHPV	2	N	Lensectomy	None	None
11	M	5 months	OD	ROP	1	N	Lensectomy +Vtx	None	None
12	F	10 months	OS	FEVR	1	N	Lensectomy +Vtx	Iris incarceration	None
13	M	2 years	OD	FEVR	2	N	Lensectomy	None	None
14	F	8 months	OD	FEVR	1	N	Lensectomy+Vtx	None	None
15	F	1 year	OS	ROP	1	N	Lensectomy +Vtx	None	None
16	M	2 months	OD	FEVR	1	N	Lensectomy	None	None
17	F	1 year	OD	ROP	1	N	Lensectomy+Vtx	None	None
18	M	1 year	OS	FEVR	2	N	Lensectomy	None	None
19	F	6 months	OS	PHPV	1	N	Lensectomy+Vtx	None	None
20	F	6 months	OS	FEVR	1	N	Lensectomy+Vtx	None	None
21	M	6 months	OD	FEVR	1	N	Lensectomy	Iris incarceration	None
22	M	5 months	OS	FEVR	1	N	Lensectomy	None	None
23	M	2 years	OS	FEVR	1	N	Lensectomy	None	None
24	M	2 years	OS	FEVR	1	N	Lensectomy+Vtx	None	None
25	F	7 months	OD	FEVR	2	N	Lensectomy	None	None
26	F	2 years	OS	FEVR	1	N	Lensectomy+Vtx	Iris incarceration	None
27	F	1 year	OD	PHPV	3	Y	Lensectomy	None	None
28	M	2 months	OD	FEVR	2	N	Lensectomy	None	None
29	F	1 year	OD	FEVR	2	N	Lensectomy	None	None
30	F	1 year	OS	FEVR	2	N	Lensectomy+Vtx	None	None
31	F	1 year	OD	PHPV	2	N	Lensectomy+Vtx	None	None
32	M	3 months	OD	FEVR	1	N	Lensectomy	None	None
33	M	1 month	OD	PHPV	1	N	Lensectomy+Vtx	Iris incarceration	None
34	F	8 months	OD	FEVR	1	N	Lensectomy+Vtx	Iris incarceration	None
35	M	2 months	OS	FEVR	3	Y	Lensectomy	None	None
36	M	6 months	OS	FEVR	1	N	Lensectomy+Vtx	None	None
37	F	1 year	OS	FEVR	1	N	Lensectomy+Vtx	None	None
38	M	2 months	OS	FEVR	1	N	Lensectomy	None	None
39	M	9 months	OD	FEVR	1	N	Lensectomy	None	None
40	M	7 months	OS	ROP	1	N	Lensectomy+Vtx	None	None
41	M	7 months	OD	FEVR	1	N	Lensectomy	Iris incarceration	None

FEVR, familial exudative vitreoretinopathy; ROP, retinopathy of prematurity; PHPV, persistent hyperplastic primary vitreous; Vtx, vitrectomy; M, male; F, female; OD, right eye; OS, left eye; N, none; Y, yes.

## Discussion

Advanced pediatric vitreoretinopathies tend to be more aggressive, leading to severe complications, and causing pain and opacity of the cornea (10). Angle-closure glaucoma (ACG) or angle closure is a rare clinical condition in children, which is always a secondary condition, and the possible causes included anterior displacement of the lens-iris diaphragm due to retrolental fibrovascular tissue contraction, pupillary block, and ciliary block (11, 12). It was hard to separate the iris or/and the lens from the corneal endothelium with viscoelastic; the first step was to lower the IOP. At this time, the viscoelastic can be easily injected into the AC, separating the capsule-endothelial, iris-endothelial, capsule-iris adhesion, and reconstructing the AC. Iris puncture helped to offer a chance for lensectomy with anterior vitrectomy or vitrectomy and provide chances to open the anterior chamber angle.

**FIGURE 5:**
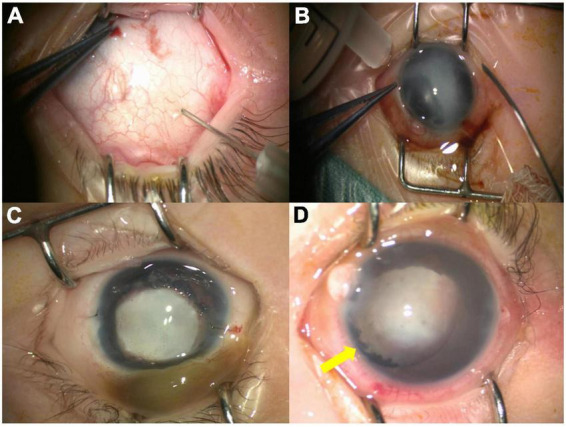
The disadvantages of external drainage of subretinal fluid (SRF). Subretinal fluid external drainage **(A)**. The constant outflow of SRF may affect the surgical maneuvers and decrease the definition of surgical field **(B)**. The constant outflow of SRF can cause the subconjunctival effusion **(C)**. The iridodialysis (yellow arrow) can be caused by the sudden rapid outflow of SRF **(D)**.

Traditionally, external drainage of SRF was indicated for the achievement of anterior chamber formation in cases with flat anterior chamber (3, 4, 6). However, it might be complicated by hemorrhage, incarceration of the retina, and/or vitreous or retinal perforation (6, 7). Except for these conventional complications, the author’s personal experience showed that sudden rapid outflow of SRF may lead to iridodialysis. In addition, in the process of conducting scleral depression, the subconjunctival effusion produced by the constant outflow of SRF would reduce the clarity of the surgical field (Figure 5).

In advanced pediatric vitreoretinopathy with total retinal detachment, the disappearance of the anterior chamber can be generated by the fronted lens and iris. In such cases, except for the SRF drainage, releasing the aqueous humor in the posterior chamber can also contribute to the formation of the anterior chamber and create chances for further maneuvers. Thus, inspired by the iridotomy, the authors make a creative usage of the iris puncture to assist the formation of AC in patients caused by advanced vitreoretinopathies, which naturally avoid the external SRF drainage-related problems. The usage of iris puncture has several advantages over the external drainage of SRF. First and most importantly, the iris puncture through the corneal incision is effective in the formation of AC. In most cases, AC formation can be achieved through a maximum of two episodes of iris puncture. Second, the usage of iris puncture simplifies the surgical procedures, thereby shortening the total operative time. Third, the puncture site (s) was (were) designed according to the further surgical maneuvers. Thus, no extra incisions were demanded in subsequent operations. Fourth, fewer complications occurred. In this study, the only intraoperative complication was iris incarceration, which could be easily corrected, and no other related postoperative complications were found in any cases. Fifth, the iris puncture, unlike external drainage of SRF, would not affect the surgical vision field. Last, this technique is simple with a short learning curve. The AC formation was achieved in 95.1% of eyes in our study *via* a simple iris puncture technique. The combination of external drainage of SRF was performed if the anterior chamber formation failed after three episodes of iris puncture. At the last visit, all eyes had a reconstructed AC.

In conclusion, the creative iris puncture technique is effective, simple, safe, and easy to master in the management of the disappeared anterior chamber caused by various advanced pediatric vitreoretinopathies. Compared to the traditional external SRF drainage, this technique simplifies surgical procedures and decreases the incidence of surgical complications.

## Data availability statement

The raw data supporting the conclusions of this article will be made available by the authors, without undue reservation.

## Ethics statement

The studies involving human participants were reviewed and approved by Ethics committee of Xinhua Hospital Affiliated to Shanghai Jiaotong University. Written informed consent to participate in this study was provided by the participants’ legal guardian/next of kin.

## Author contributions

CC wrote the manuscript. FH drew the figures. TT and PF collected the data. YC revised the manuscript. PZ provided the conception. All authors contributed to the article and approved the submitted version.
